# Surgical interhospital transfer mortality: national analysis

**DOI:** 10.1093/bjs/znad042

**Published:** 2023-03-01

**Authors:** Ishraq Murshed, Aashray K Gupta, Angelique N Camilos, Ahad Sabab, Stephen Bacchi, Joshua G Kovoor, Justin C Y Chan, Guy J Maddern

**Affiliations:** Discipline of Surgery, University of Adelaide, Adelaide, South Australia, Australia; Discipline of Surgery, University of Adelaide, Adelaide, South Australia, Australia; Department of Cardiothoracic Surgery, Gold Coast University Hospital, Southport, Queensland, Australia; Discipline of Surgery, University of Adelaide, Adelaide, South Australia, Australia; Discipline of Surgery, University of Adelaide, Adelaide, South Australia, Australia; Discipline of Surgery, University of Adelaide, Adelaide, South Australia, Australia; Discipline of Surgery, University of Adelaide, Adelaide, South Australia, Australia; Royal Australasian College of Surgeons, Adelaide, South Australia, Australia; Discipline of Surgery, University of Adelaide, Adelaide, South Australia, Australia; Department of Cardiothoracic Surgery, New York University Langone Health, New York, NY, USA; Discipline of Surgery, University of Adelaide, Adelaide, South Australia, Australia; Royal Australasian College of Surgeons, Adelaide, South Australia, Australia

## Abstract

**Background:**

Interhospital transfers of surgical patients are an independent risk factor for mortality. The Australian and New Zealand Audit of Surgical Mortality (ANZASM) aims to improve surgical care through assessment of all cases of surgical mortality. This study aimed to describe common clinical management issues that contributed to interhospital transfer patient mortality.

**Methods:**

Data for all surgical patient mortality in Australia (except New South Wales) that underwent interhospital transfer between 1 January 2010 and 31 December 2019 were extracted from ANZASM. The surgeons’ reports and assessors’ evaluations were examined to identify clinical management issues. Thematic analysis was performed to develop pertinent themes and subthemes.

**Results:**

Some 8679 patients were identified over the 10-year period. Of these, 2171 (25.0 per cent) had 3259 clinical management issues identified. Prominent themes were operative design (*n* = 466, 14.3 per cent), decision to operate (*n* = 425, 13.0 per cent), medical conditions (*n* = 344, 10.6 per cent), diagnosis (*n* = 326, 10 per cent), transfer (*n* = 293, 10.0 per cent), intraoperative issues (*n* = 278, 8.5 per cent), inadequate assessment (*n* = 238, 7.3 per cent), communication (*n* = 224, 6.9 per cent), delay in recognizing complications (*n* = 180, 5.5 per cent), coagulopathy (*n* = 151, 4.6 per cent), insufficient monitoring (*n* = 127, 3.9 per cent), infection (*n* = 107, 3.3 per cent), and hospital resources (*n* = 100, 3.1 per cent). Assessors considered 58.4 per cent of clinical management issues (*n* = 1903) probably or definitely preventable.

**Conclusion:**

This study identified 13 themes of potentially avoidable management issues present in surgical mortality following interhospital transfers. Quality-improvement initiatives targeting these areas may improve surgical patient outcomes.

## Teaser

Interhospital transfers (IHTs) are commonplace in a patient’s healthcare journey; however, IHTs have been shown to be an independent risk factor for mortality in surgical patients. We analysed a national audit of 8679 surgical patient IHT mortality over a 10-year period to identify 13 prominent themes of potentially avoidable management issues. Quality-improvement initiatives targeting these areas may improve surgical patient outcomes.

## Introduction

Geographical factors of health networks can produce significant discrepancies in the provision of health care between metropolitan and rural hospitals. Interhospital transfers (IHTs) can facilitate the delivery of timely surgical care within large health networks and resolve issues such as lack of appropriate resources at the index location, higher acuity of care requirement, or need for complex multidisciplinary specialist care^1,2^.

IHTs have been demonstrated to be an independent risk factor for increased mortality in many surgical cohorts^3–6^. These factors can be categorized into patient, disease, and transport factors. Patient factors include older age and lower socio-economic background, while disease factors include level of complexity and severity^3–5^. Transport factors that can lead to potential delays in definitive surgical care include method of transportation and distance travelled^3–5^. IHT patients have higher in-hospital mortality and healthcare costs, and poorer outcomes than their non-transferred counterparts^6^. Given that IHT is associated with 1 in 13 hospital admissions in Australia^7^ and entails considerable cost^6^, it is imperative to ensure that they are conducted effectively to optimize patient benefit.

With its large land area and widely dispersed population, Australia provides an example of a setting within which health networks must overcome geographical issues to provide equitable care to metropolitan and rural centres. The Australian and New Zealand Audit of Surgical Mortality (ANZASM) is a national, independent, peer-reviewed surgical audit overseen by the Royal Australasian College of Surgeons^8^. Since its inception, it has identified major issues for intervention in surgical care of patients by aggregating national surgical outcomes^9^. To highlight potentially avoidable issues and facilitate quality improvement, this study examined 10 years of Australian mortality data on surgical patients who were transferred between hospitals and aimed to identify common clinical management issues that contributed to patient mortality. Highlighting potentially avoided clinical management issues from these deaths could be used as a foundation for future quality-improvement strategies and surgeon education to enhance patient care.

## Methods

### ANZASM data collection

All surgical patient mortalities in Australia are reported to ANZASM^8^. ANZASM is notified of a patient’s death by the Department of Health and the medical records department of the participating hospital^8,10^. At present, 99 per cent of Australian surgeons and 100 per cent of public and private hospitals performing surgery participate in the audit^10^. Since 2010, all fellows of the Royal Australasian College of Surgeons are mandated to participate in the mortality audit and this is required for re-certification^10^. Following notification of the death of a patient for whom a surgeon had cared for, or had significant involvement with, a standardized surgical case form is completed by the treating surgeon and returned to ANZASM^8^. The surgical case form return rate for participating surgeons is 92 per cent^10^. The surgical case form is deidentified and peer reviewed by an independent surgeon from the same specialty, but different hospital, for first-line assessment. If the assessor determines that there was adequate data to provide assessment regarding the quality of care and highlight areas of improvement, then the case is closed, with no further investigation warranted. However, if the first-line assessment determines that the data for assessment are inadequate, or specific aspects of care require further investigation, a second line assessment by an additional independent reviewing surgeon from the same specialty is organized, with the addition of information from the original medical records. The assessor is able to comment on aspects of patient management that may have been improved or contributed to patient mortality, deemed clinical management issues. These were then classified into three levels of seriousness: area for consideration, area of concern, or adverse event^10^. Each clinical management issue was also assessed for clinical impact and preventability. Clinical impact was assessed on a 3-point scale from ‘made no difference’ to ‘caused death of a patient otherwise expected to survive’. Similarly, preventability was assessed on a 4-point scale from ‘definitely not preventable’ to ‘definitively preventable’. The independent assessor’s report is provided to the treating surgeon.

### ANZASM data extraction

Patient demographics and assessor reports for patients that had an IHT were extracted from the ANZASM database. This included all surgical patient mortality in Australia (except New South Wales) over a 10-year period between 1 January 2010 to 31 December 2019. Cases prior to 2010 were excluded owing to the risk of reporting bias, since participation was not mandatory^8^. Cases in which there were incomplete data were excluded from qualitative analysis and characterization of clinical management issues.

### Qualitative analysis

Surgical case form and assessor report data were examined for clinical management issues for all patients. The available narrative reports from second-line assessment and clinical management issues identified by the ANZASM assessors were qualitatively analysed using a thematic analysis technique with an inductive data-driven approach, developed based on methodology described by Braun and Clarke^11^. This method of thematic analysis has been used to examine trends of clinical management issues in patient mortality from ANZASM data in endocrine surgery, neurosurgery, urology, and cardiothoracic surgery^12–15^. All clinical management issues were analysed regardless of level of seriousness, and each patient case may have contained one or more clinical management issue. Patients were included for analysis irrespective of whether the assessor deemed the death as preventable, as clinical management issues that occur in unavoidable deaths can still provide important lessons.

Two independent authors (I.M., A.N.C.) each reviewed all assessor reports, noting key ideas and issues identified in clinical management issues. Initially, 100 cases were coded by the two authors and an initial coding framework developed in consultation with other senior authors. Discrepancies in the initial coding framework were resolved by discussion among the authors. After finalizing this coding framework, two independent authors (I.M., A.N.C.) coded the data across the entire data set in a systematic fashion, collating data relevant to each code, with overall agreement of 82 per cent. Any discrepancies in the final coding were resolved by evaluation by a third author (A.S.). Following this, the coding framework was collated to construct and define potential themes. The themes were reviewed by the authors to ensure appropriateness in relation to each coded data extract, as well as the entire data set, producing a thematic ‘map’ of the analysis. After reviewing and refining of the themes within the coding framework, subthemes were explored to develop the finalized thematic analysis of the available qualitative data set.

## Results

**Fig. 1:**
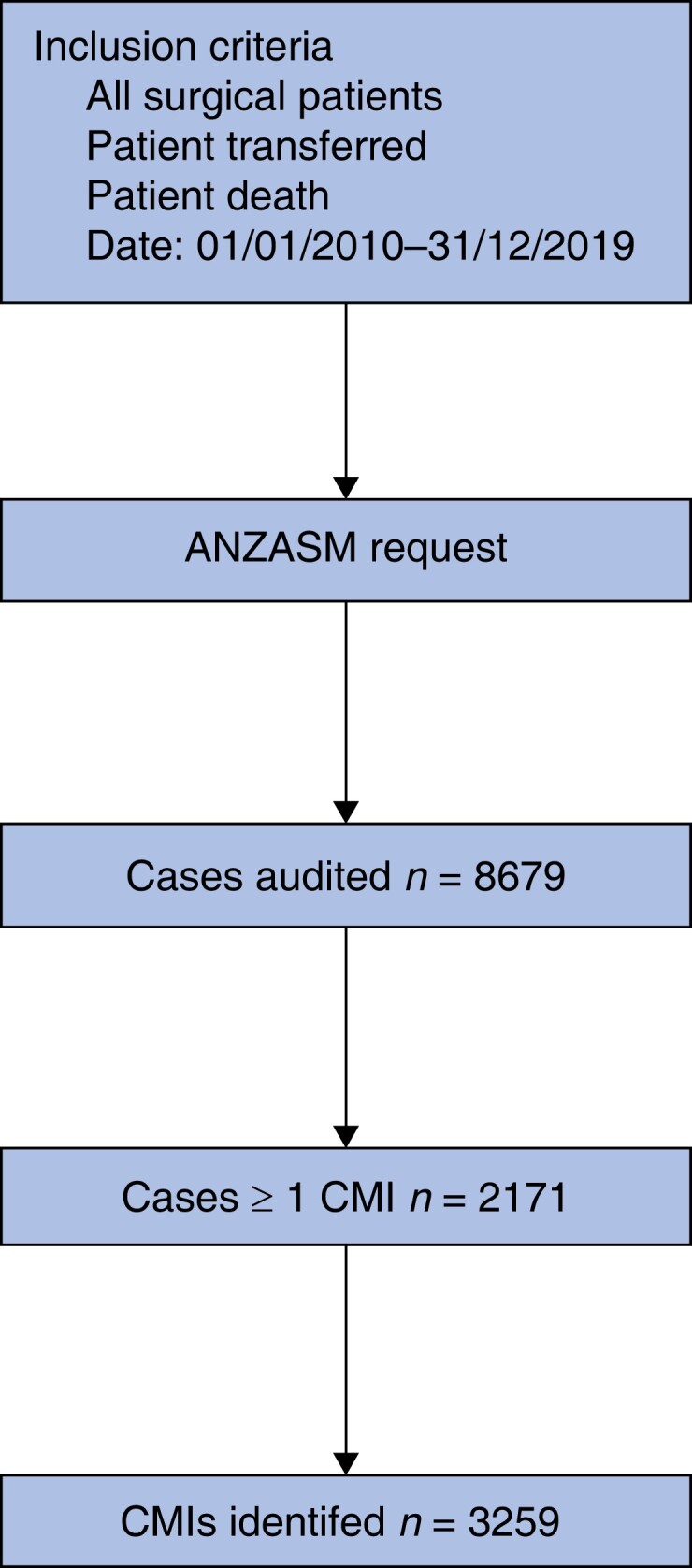
Flowchart of cases audited by the Australian and New Zealand Audit of Surgical Mortality with inclusion criteria, between 1 January 2010 to 31 December 2019, and the number of clinical management issues (CMIs) identified

In the 10-year period between 1 January 2010 and 31 December 2019 there were 8679 cases of surgical mortality audited by the ANZASM where the patient underwent an IHT (*Fig. 1*). Of these, 2171 (25.0 per cent) patients were identified to have at least one clinical management issue. Cases were resolved after first-line assessment in 1260 (58.0 per cent) of cases, with the remaining 911 cases (42.0 per cent) resolved after the second-line assessment. Of the 2171 patients identified that had at least one clinical management issue, 2032 (93.6 per cent) were emergency hospital admissions, 119 (5.5 per cent) elective hospital admissions, and 20 (<1 per cent) patients with missing data.

### Characterization of clinical management issues

**Fig. 2:**
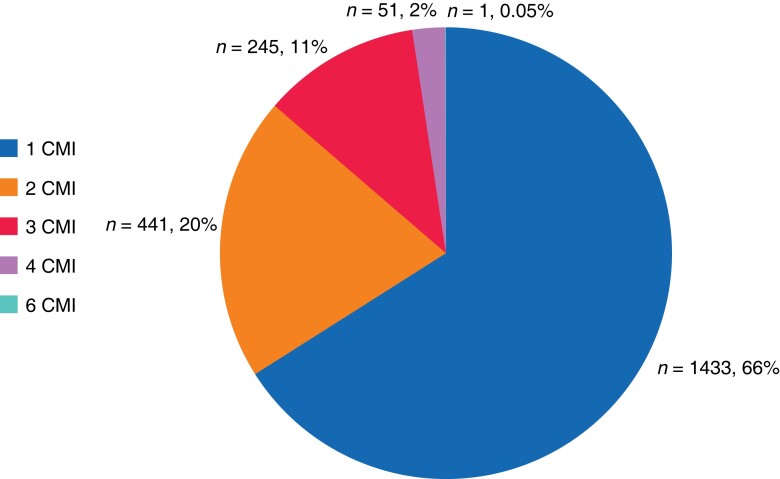
Percentage of interhospital transfer mortality cases with at least one clinical management issue (CMI)

Of the 2171 patients, 3259 clinical management issues were identified that may have contributed to patient mortality. In total, 1433 (66.0 per cent) patients had one clinical management issue identified, 441 (20.3 per cent) had two, 245 (11.3 per cent) had three, 51 (2 per cent) had four, and one patient had six clinical management issues identified (*Fig. 2*).

**Fig. 3:**
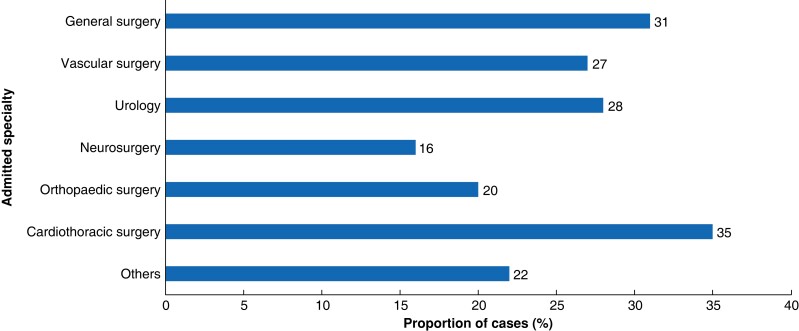
Proportion of interhospital transfer mortality cases with at least one clinical management issue by admitted specialty

In terms of surgical specialty of admission for clinical management issues in IHT mortality, the most common was general surgery (830 patients; 38.2 per cent), followed by cardiothoracic surgery (346 patients; 15.9 per cent), neurosurgery (339 patients; 15.6 per cent), orthopaedic surgery (266 patients; 12.3 per cent), vascular surgery (225 patients; 10.4 per cent), urology (67 patients; 3.1 per cent) and other surgical specialties (85 patients; 3.9 per cent). The proportion of patients with clinical management issue *versus* total IHT mortality patients sorted by surgical specialty of admission is shown in *Fig. 3*.

Clinical management issues were graded in terms of seriousness. In total, 1851 (56.8 per cent) clinical management issues were identified as an area of consideration that the assessor believed could have been improved, 999 (30.7 per cent) were an area of concern, and 369 (11.3 per cent) were unintended adverse events or injury caused by medical management which led to prolonged hospitalization, patient impairment, or death. Forty (1.2 per cent) clinical management issues had incomplete data collection for seriousness and were not included.

Clinical management issues were graded for clinical impact to determine whether they contributed to patient death. In total, 971 (29.8 per cent) clinical management issues were considered to have made no difference to patient death, 1917 (58.8 per cent) may have contributed to death, and 255 (7.8 per cent) were considered to have caused the death of patients who would otherwise have been expected to survive. Altogether, 116 (3.6 per cent) clinical management issues had incomplete data collection for clinical impact and were not included.

Clinical management issues were graded by assessors for preventability. In total, 146 (4.5 per cent) clinical management issues were deemed definitely unpreventable and 1003 (30.8 per cent) were deemed probably unpreventable. Assessors deemed that 1256 (38.5 per cent) clinical management issues were probably preventable and 647 (19.9 per cent) were deemed to be definitely preventable. Altogether, 207 (6.4 per cent) clinical management issues had incomplete data collection for preventability and were not included.

### Thematic analysis

**Fig. 4:**
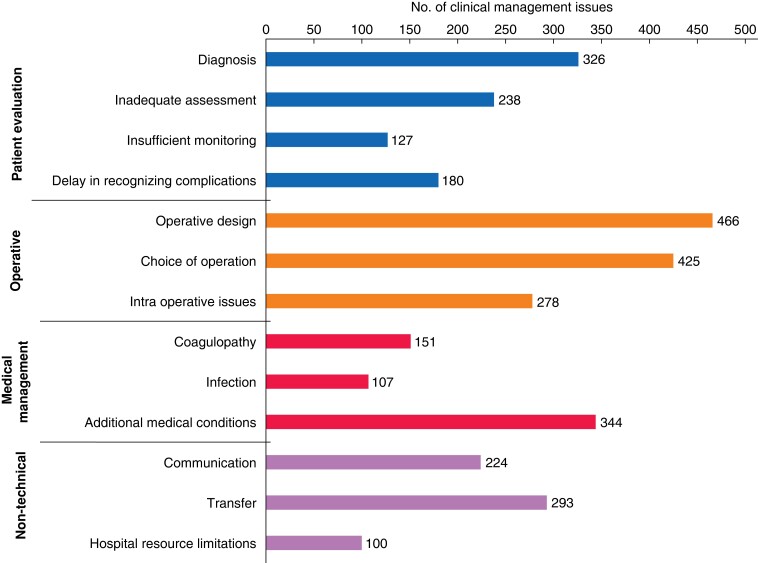
All interhospital transfer mortality clinical management issues by theme

Clinical management issue analysis revealed trends in four key domains of patient care: patient evaluation (871 issues; 26.7 per cent), operative (1169 issues; 35.9 per cent), medical management (602 issues; 18.5 per cent), and non-technical (617 issues; 18.9 per cent) (*Fig. 4*). Key themes identified in the evaluation of patients included diagnostic issues, a delay in recognizing complications, inadequate assessment, and insufficient monitoring. In the operative domain, key themes identified were the decision to operate, operative design, and intraoperative problems. In the medical management domain, prominent themes were inappropriate management of coagulopathy, infection, and patient’s additional medical conditions. Finally, prominent themes in the non-technical domain identified were issues in communication, patient transfer, and hospital resource limitations. Subthemes were identified in each major theme (*Table 1*).

**Table 1:** Thematic analysis of clinical management issues in 3259 interhospital transfer surgical patients audited by the Australian and New Zealand Audit of Surgical Mortality from 1 January 2010 to 31 December 2019

Major themes	No. of cases (*n*)	Major subthemes	No. of cases (*n*)	Case example
**Patient evaluation (*n* = 871)**				
ȃDiagnosis	326	Incorrect initial diagnosis	74	Large abdominal aortic aneurysm diagnosed as renal colic
		Diagnosis missed or unidentified	33	Missed diagnosis of oesophageal perforation at index hospital
		Delay to reaching correct diagnosis	219	Delayed diagnosis of urosepsis for 48 h until patient admitted to ICU with multiorgan failure
ȃInadequate assessment	238	Preoperative optimization	65	Preoperative optimization of hyperglycaemia, electrolyte abnormalities, and fluid balance
		Delay in assessment of patient	16	Admitted for 2 days with closed loop small bowel obstruction prior to surgical review
		Inappropriate or delay in imaging	52	CT of the head not performed after a fall with head-strike in a patient with INR 5.0
ȃInsufficient monitoring	127	Failure to use ICU	49	Not utilizing ICU for patient with severe pancreatitis with several comorbidities
		Early discharge	14	Discharged from hospital while awaiting major surgery in which time the patient deteriorated
		Inappropriate level of nursing care	27	Patient had a fall in the hospital 12 days postoperatively and developed a subdural haematoma
ȃDelay in recognizing complications	180	Anastomotic leak	26	Developed anastomotic leak 6 days after extended right hemicolectomy and not recognized until day 12
		Bleed	19	Undetected right thoracic postoperative bleeding following CABG manifesting as anaemia
		Ischaemic event	19	Delay in recognizing ischaemic leg after emergency repair of ruptured AAA
		Infection	20	Delay in recognition of sepsis secondary to diabetic foot infection by the overnight medical officer
**Operative (*n* = 1169)**				
ȃDecision to operate	425	Inappropriate choice	288	Exploratory laparotomy after CT evidence of viscus perforation in a comorbid 89-year-old patient
		Uncertain indication	137	Fixation of femoral fracture in patient with significant comorbidities and not palliative therapy
ȃOperative design	466	Timing of operation	157	Performing high-risk spinal surgery outside daylight hours for a multimorbid elderly patient
		Delay to operation	107	Delay of 9 h between diagnosis of intracerebral haemorrhage and operative decompression
		Different operation suggested	202	Colectomy and primary anastomosis in perforated feculent diverticulitis without covering stoma
ȃIntraoperative issues	278	Operative complications	80	Pulmonary cement embolism during cemented hemiarthroplasty for neck of femur fracture
		Iatrogenic injury	138	Injury to pancreatic arterial supply during laparoscopic surgery resulting in pancreatic necrosis
		Surgical technique	60	Aortic cannulation technique in extensively calcified aorta resulting in embolic stroke and death
**Medical management (*n* = 602)**				
ȃCoagulopathy	151	VTE prophylaxis omitted	42	DVT prophylaxis not used during admission for dislocated shoulder and patient developed pulmonary embolism
		Postoperative bleeding	49	Postoperative bleeding requiring resternotomy in ICU
		Incorrect anticoagulant medications	49	Therapeutic anticoagulation of patient with acute splenic injury with subsequent haemorrhage
ȃInfection	107	Pneumonia	47	Postoperative aspiration pneumonia
		Wound	11	Persistent wound ooze following hip hemiarthroplasty managed only on readmission
		Inappropriate antibiotics	22	Empiric antibiotics not provided when cholangitis was provisionally diagnosed at admission
ȃMedical conditions	344	Fluids and haemodynamic function	81	Inadequate fluid replacement with transfer delays causing dehydration and AKI
		Unsatisfactory postoperative care	25	No medical consultation for postoperative chest pain and troponin elevation following laparotomy
		Misuse of medications	29	Withholding usual therapeutic anticoagulant for 5 days after colonoscopy resulting in ischaemic gut
**Non-technical (*n* = 617)**				
ȃCommunication	224	Documentation	89	Lack of documentation from treating surgeon in hospital notes regarding ongoing management plan
		Handover	64	Failure to communicate change in clinical status by referring team to the receiving team during transfer
		Leadership	58	Lack of leadership in patient management when multiple teams contributing to patient care
ȃTransfer	293	Delay in transfer	215	Delayed transfer for 40 h for a patient with large bowel obstruction prior to operative intervention
		Inappropriate indication for transfer	72	Transfer of patient to tertiary hospital for surgery when patient did not want surgical intervention
		Complications during transfer	6	Inadequate airway protection during transfer with two aspiration events leading to severe pneumonia
ȃHospital resource	100	Inadequate staffing	71	Offsite radiology reporting at index hospital, with undiagnosed perforated diverticulitis
		Shortage of appropriate hospital beds	12	Delay in decompressive craniotomy due to lack of beds available in neurosurgery centre
		Resources unavailable or delayed	17	Cell saver autotransfusion device not available in major regional hospital during emergency repair of an AAA

ICU, intensive care unit; INR, international normalized ratio; CABG, coronary artery bypass grafting; AAA, abdominal aortic aneurysm; VTE, venous thromboembolism; DVT, deep vein thrombosis; AKI, acute kidney injury.

## Discussion

This 10-year analysis of national surgical mortality data identified key issues for IHT patients. Two main groups of patients requiring IHT were identified, both of which are different and therefore clinical management issues represented distinct differences. The first group is emergency IHT (often from the emergency department) due to an unstable patient with a surgical diagnosis that cannot be treated locally. The second group comprises patients that have been diagnosed and are waiting for specialist care unavailable at the index location, such as neurosurgery or cardiothoracic surgery, and receive their initial radiology and work-up in the regional hospital but require transfer to a larger metropolitan service for their definitive management. These may inform clinical modifications at an individual hospital, health network, and broader systems level that could significantly reduce mortality associated with surgical IHTs. Further, the identified themes may facilitate the production of effective quality- and safety-improvement initiatives.

Patient evaluation forms the foundation for recognition of deteriorating patients and transfer of patients to an appropriately resourced hospital. Accounting for over a quarter of all clinical management issues, assessors noted diagnostic challenges, inadequate assessment, a delay in recognizing complications, and insufficient monitoring as key issues. A significant challenge is a correct and timely diagnosis, as this forms the cornerstone of efficacious management. Delay or incorrect diagnosis has a ‘domino effect’, with potential knock-on worsening of operative and postoperative outcomes, as well as increased mortality. Surgical emergencies such as aortic dissections, vascular ruptures, and septic perforations are time sensitive, and may explain why surgical specialties such as cardiothoracic surgery, vascular surgery, and general surgery have a high proportion of clinical management issues. Outcomes for these conditions depend on early diagnosis and surgical treatment, and delay in achieving this increases mortality proportional to the time delay. The development and implementation of clear protocols and guidelines for clinical assessment and investigation may reduce the potential for diagnostic error^16^. Clinical pathways for specific emergency high-risk surgical conditions, which identify presenting symptoms and suggest high-yield investigations to delineate between serious conditions, could be used to improve patient safety. A 2010 Cochrane review identified clinical pathway documents that link up-to-date evidence with specific health conditions are associated with reduced in-hospital complications and improved documentation without negatively affecting duration of hospital stay or cost^17^. A recent analysis of IHT patients using ANZASM data found that inadequate clinical assessment resulted in a 49.5-fold increased likelihood of transfer delay^18^, resulting in poorer patient outcomes^4^. Finally, important clinical management issues were identified associated with insufficient monitoring and a delay in recognizing complications. Adverse events are often predicted by observable physiological and clinical abnormalities^19^, and early identification of these signs of deterioration can improve outcomes^20^. Standardized early-warning systems and medical emergency teams have been implemented^21^ to address this, and remains an area of ongoing national review and action in Australia^22^. Artificial intelligence algorithms may also have an increasing role in the detection of patient deterioration^23^.

Many operative clinical management issues were identified, with key areas of concern being the decision to operate, operative planning, and intraoperative complications. Most concerns in this group were related to clinical decision-making regarding decision to operate and operative planning. In this patient cohort, during the first operation (1929 patients), the consultant surgeon was responsible for the decision to operate and operative planning in 92 per cent of cases, comparable to the 90 per cent attendance previously reported using national Australian data analysing postoperative deaths^24^. This suggests satisfactory rates of consultant involvement, and achieving a higher rate in Australia may not be feasible with senior registrars performing minor operations in many hospitals^25^. The high proportion of incidents coupled with high rates of consultant involvement may suggest a role for increased consultation among senior clinicians and multidisciplinary team discussions regarding complex patients. Increased use of telehealth services to provide clinical assistance in regional areas may also be of benefit^26^. Finally, intraoperative events were defined as the culmination of surgical technical challenges and other intraoperative complications. This may be thought of as a summation of preoperative factors that preceded the sentinel operative incidents. Additionally, as the vast majority of cases were consultant-led with expected adequate technical expertise, the role of non-technical errors inside the operative theatre requires consideration^27^. Continued education and training for all surgeons in non-technical skills may be beneficial in ameliorating this.

Management of medical issues was present in 18.5 per cent of clinical management issues. Key themes identified were management of coagulopathy, infection, and fluid therapy. Venous thromboembolism management is of specific interest as failure of appropriate prophylaxis may result in death secondary to pulmonary emboli. There has been considerable work in this field to improve patient outcomes, and national guidelines have been developed to promote appropriate practice^28^. The average age in the examined patient cohort was 70 years, with multiple medical comorbidities and heterogeneous medical management issues identified. Data from orthopaedic surgery have identified a co-management model with medical specialties significantly improved mortality following hip fractures^29^. Given the elderly, multi-comorbid and high-risk population of surgical IHT patients, a similar model could be hypothesized to improve patient mortality by holistically addressing baseline medical conditions in addition to the emergency surgical problem. Additionally, higher utilisation of intensive care unit (ICU) resources upon transfer may be beneficial. Insufficient monitoring was a key theme in issues identified in patients who are transferred due to need for higher level of care from patient or disease factors, and these cases may warrant earlier ICU review and support.

Non-technical factors such as communication issues, delays in transfer, and hospital resource limitations accounted for 18.9 per cent of all clinical management issues identified. Good communication is essential for prompt and appropriate transitions of care^22^, as well as successful management following transfer. Harl *et al.* showed that a significant number of transfer notes in emergency general surgery patients were missing crucial clinical details, as well as radiological reports^30^. Poor communication has been associated with a greater than sixfold increase in the likelihood of delay in transfer^18^. As such, standardization of information exchange during transfer using a structured checklist may improve interhospital efficiency and patient safety. Widespread use of electronic medical records may also aid in improving communication and handover during transfer; however, the use of a consistent system over multiple institutions remains a challenge. In the Australian state of Victoria all IHTs due to trauma is coordinated through Adult Retrieval Victoria, a centralized retrieval system that has access to the on-call schedule and bed availability of all metropolitan ICUs and can rapidly facilitate a conference call between all the specialists needed for case discussion. Using a centralized retrieval team may help to ameliorate the challenges in communication during IHT, and has been shown to reduce mortality and improve long-term patient outcomes^31^. Most transfer-related clinical management issues were due to a delay in transfer. A recent systematic review by Young *et al.* revealed that there remains a general paucity of knowledge regarding the factors influencing IHT delays^32^. A retrospective cohort study demonstrated modifiable predictors of IHT delay were inadequate clinical assessment, poor communication and multiple transfers^18^, similar to the highlighted themes identified during this thematic analysis.

A key strength of the ANZASM assessment is the speed of review of the case information, often the complete medical records, within a matter of weeks after the death^8^. Findings may inform surgical training and clinical education at an institutional level, as well as recommendations and priorities for safety initiatives at a systemic level. Pertinent cases are highlighted regularly online in case review booklets to allow lessons to be communicated among the surgical community and not limited to the treating surgeon or institution^33^. Factors identified and influencing care in the transfer of patients in Australia may or may not be relevant in other health jurisdictions. This large national heterogenous cohort of patients includes transfers of varying urgencies, distances, settings, and demographics. This may offer potential comparisons to international settings and services.

The data collected have been self-reported by the treating surgeons, which increases bias. Additionally, assessors themselves may also introduce their own degree of bias. The effect of this bias is minimized as assessors were independent and blinded. It is possible that not all mortalities were included due to reporting bias in ANZASM; however, this is likely to have been minimized due to the high surgeon participation (99 per cent) and return of standardized case forms (92 per cent) for review and assessment. Furthermore, second-line assessment occurred in 42.0 per cent of cases allowing direct case notes review, which may have limited reporting bias of the treating surgeon. Missing data from the ANZASM may have introduced bias. This was limited in the characterization of clinical management issues as only between 1 and 7 per cent of cases had missing variables. Since all assessments of clinical management issues were performed by surgeons without input from other members of the patient’s clinical team, there may be a skew towards operative issues. Potentially, different clinical management issues may be identified if cases were to be reviewed by the medical teams or anaesthetists. As the study only encompassed mortality data and not all IHT patient data, a clear denominator for clinical management issues cannot reliably be established. Therefore, definitive prevalence values cannot be ascertained. A limitation of qualitative data analysis is that thematic generation may introduce researcher bias. Attempted minimization of this occurred by having multiple researchers examine each clinical management issue and discrepancies reviewed by a separate, independent researcher.

There remains a paucity of research examining management issues present in surgical deaths following IHT. This study analysed a large patient cohort of surgical deaths across a 10-year period over various surgical specialties in both the public and private healthcare sectors in Australia. Thirteen themes of potentially avoidable management issues present in surgical mortality following IHTs were identified. Quality-improvement initiatives targeting these areas may improve surgical patient outcomes across metropolitan and rural systems of care. Future research into this topic may involve production of a transfer safety checklist to ensure safe and effective transfers occur in addition to having a standardized method for auditing transfer performance.

## Data Availability

The data set used to conduct this research will be made available on request.
